# Challenges to Build up a Collaborative Landscape Management (CLM)—Lessons from a Stakeholder Analysis in Germany

**DOI:** 10.1007/s00267-019-01205-3

**Published:** 2019-09-25

**Authors:** Jana Zscheischler, Maria Busse, Nico Heitepriem

**Affiliations:** 1 Leibniz Centre for Agricultural Landscape Research, Research Area Land Use and Governance, Eberswalder Str. 84, 15374 Müncheberg, Germany; 2 Centre for Technology and Society (ZTG), Technische Universität Berlin, Hardenberg Str. 16-18, 10623 Berlin, Germany; 3 UNESCO Biosphere Reserve Spreewald, Schulstraße 9, 03222 Lübbenau, Spreewald Germany

**Keywords:** Integrated landscape approach, Transdisciplinary research, Tourism, Nature conservation, Cultural landscape, Land use conflict

## Abstract

Traditional cultural landscapes are of special value not only for reasons of nature conservation and high species diversity but also because they intersect with the identity of local communities, support recreation and tourism, and preserve cultural heritage. Structural changes in rural areas threaten these unique sceneries and environments in Europe and worldwide. As a result, the question of how to maintain and manage cultural landscapes where economic benefits are not assured has become a priority in science and in practice. Considering this context, community-based collaborative landscape management (CLM) can be considered an innovative and promising approach. This paper presents results from a stakeholder analysis examining the preconditions and opportunities for initiating a CLM in the biosphere reserve known as ‘Spreewald’. The results indicate that due to the type of problem (landscape change)—which is characterised by complexity, beneficial linkages to a multitude of actor groups, and broad problem awareness—CLM appears to be feasible. However, other preconditions related to social relationships among actor groups, questions of legitimate coordination and the collaborative capacity of the community are not met, thus reducing the likelihood of success. To address these challenges, we discuss the potential of transdisciplinary processes (TD) to assist local communities in establishing such a collaborative problem-solving and management approach. We show that TD is highly valuable and supportive during this critical stage of emerging collaboration.

## Introduction

Landscapes in European rural areas are experiencing ‘massive and rapid changes’ due to demographical, technological, cultural, and economic developments (Verburg et al. 2006; Antrop 2006; Agnoletti 2014). The resulting structural changes lead to widespread farmland abandonment and particularly threaten traditional cultural landscapes, which are regarded as being in a state of ‘profound transition’ (Van Eetvelde and Antrop 2004, Agnoletti 2014). These typical landscapes are often characterised by unique agricultural systems that developed under low-intensity agriculture practices highly adapted to site-specific requirements. Today, these low-intensity agricultural practices have become economically inefficient.

Still, worldwide, traditional cultural landscapes are unique sceneries and environments; they often have high biodiversity (species diversity) that results in high value for nature conservation (e.g., Luoto et al. 2003; Plieninger et al. 2006; Beilin et al. 2014). In addition, both local communities and tourists often have a strong sense of identification with these landscapes (Antrop 1997). Moreover, Agnoletti (2014) emphasises that the cultural heritage values of Europe’s historical landscapes may reach far beyond local interests as these landscapes are cultural products documenting ‘past civilisations’ and representing ‘the genius of their builders’ (p.67). Against this backdrop, the issue of how to maintain and manage cultural landscapes, when cultivation is no longer profitable, has become a priority question among scientists, policy makers and practitioners in the field of land use science (Hernández-Morcillo et al. 2017).

It has been recognised that the conservation of cultural landscapes is hampered by the lack of adequate policies that put an emphasis on the protection of cultural heritage (e.g. typical housing, landscape structure). Even if many cultural landscapes are part of protected areas, dominant conservation goals often favour renaturalisation and ‘degradation of historical landscapes’ (Agnoletti 2014). In addition, maintenance measures to conserve landscape scenery and biodiversity are time-consuming and expensive. Thus far, a huge funding gap has prevented the adequate financing of nature conservation and the minimisation of biodiversity loss (Parker et al. 2012).

In this context, there is an urgent demand for new solutions and innovations to help manage landscapes sustainably. However, questions also arise concerning the normative objectives that are guiding the development and management of such landscapes: is the mere conservation and archiving of traditional landscapes reasonable? How can such landscapes be developed in a way that maintains their specific characteristics and sites with high natural value and at the same time provides just and equal benefits for different concerned actors?

As land(scape) use issues are characterised by complex actor-constellations, conflicting interests and demands as well as many sustainability problems, there is a need for integrated solutions that combine ecological, economic and social benefits. In this context, several authors emphasise the roles and opportunities of collective action and collaborative community initiatives for sustainable landscape management (e.g., Enengel et al. 2011, Prager et al. 2012, Hernández-Morcillo et al. 2017, Leach et al. 1999, García-Martín et al. 2016, Scherr et al. 2013). Frequently mentioned benefits of such approaches include the following: tackling challenges and opportunities for landscape stewardship more effectively and pro-actively compared with single actors (Scherr et al. 2013); the emergence of creative solutions (Fadeeva 2005); sharing and mobilising resources (Cong et al. 2014); negotiating and harmonising conflicting objectives; building capacity and social capital, resulting in mutual appreciation and support (Prager et al. 2012, 2015); increased knowledge exchange and communication; and engagement with the landscape and countryside (Franks and McGloin 2007). In sum, collaborative approaches are widely acknowledged to support landscape management because they are adaptive and can be tailored to site-specific conditions. In addition, they improve legitimacy and effectiveness in decision-making (e.g., Berkes 2002, Enengel et al. 2011, Olsson et al. 2004, Loft et al. 2015) and can reduce institutional misfit.

However, collaborative approaches to landscape management also face a series of challenges, such as ‘the dilemma between individual and collective benefits’, ‘trade-offs between different objectives’ (Prager 2015, p. 62) and unbalanced power relations (Almeida et al. 2018). Collaboration creates higher transaction costs, which can be unequally distributed (Enengel et al. 2011, Prager 2015). Collaboration is also dependent on the willingness of actors to contribute to and invest time in a project (Höppner et al. 2008, Enengel et al. 2011, Prager 2015, Almeida et al. 2018). Furthermore, the need for suitable organisational structures, the prerequisite of building trust and social capital, and whether a group has the maturity required to collaborate are emphasised as important factors for successful collaboration (e.g., Evans et al. 2011, Trimble and Berkes 2013, Almeida et al. 2018).

The principles of community management (collective action or co-operation) are well described in the context of common pool resources (e.g., Ostrom 1990; Cox et al. 2010) and are confirmed by a multitude of case studies (e.g., Evans et al. 2011, Faehnle and Tyrväinen 2013, Sattler et al. 2015, Almeida et al. 2018). However, it is also acknowledged that site-specific settings can differ widely due to specific local constellations of actors and institutional functions. Accordingly, existing approaches are very diverse (e.g., Sattler et al. 2015, Ostrom 2001, Pahl-Wostl 2009). Thus, there is a multitude of case studies that not only consider very different types of natural resource systems such as fisheries, water, and forests but also focus on many different aspects of collaboration (e.g., Ostrom 1990, Cox et al. 2010).

Most case studies address established resource use systems but do not answer the question of how these ‘regimes’ have evolved. Bürgi et al. (2017) found that there are ‘only very few documented examples of practical implementation’. The preconditions of collaborative resource management and the processes by which these collaborative approaches emerged have rarely been studied (Berkes 1997, Plummer and Fitzgibbon 2004). In the context of collaboration, one can assume that social relationships and mechanisms play a major role. Although the importance of this topic has been emphasised for a long time (Pinkerton 1989), there has been limited attention to the interrelations between the involved actors. In addition, while most studies address the risk and management challenges of resource overexploitation, traditional cultural landscapes are often affected by the abandonment of land use. Yet, the intertwined issues of farmland abandonment and loss of traditional cultural landscapes have not received much attention. In our literature review, we found only a few examples of studies focusing on this topic, including case studies from wetland abandonment in Sweden (Biggs et al. 2010), the biosphere reserve of the Swabian Alb in Germany (Plieninger et al. 2013), and traditional grasslands in Great Britain (McGinlay et al. 2017).

Still, empirical evidence is needed ‘to identify key challenges, opportunities, and lessons learnt’ (Loft et al. 2015, p. 150). A critical question remains open: why do some collaborations succeed while others fail? We assume that the way local actors shape their exchange relations plays a decisive, but so far neglected, role. As shown by the meta-analysis of Evans et al. (2011), more emphasis has been placed on questions of institutional settings than on social mechanisms and human dimensions.

To address this research gap, this paper presents the results of a case study of a traditional cultural landscape in North-eastern Germany. The area of focus is the Spreewald region, which is very popular for its unique landscape and cultural heritage.

The objective of this study is to better understand how we can build up collaborative landscape management (CLM) that successfully develops and maintains traditional cultural landscapes. Therefore, we sought to gain insights into the motives and roles of actors, their interactions, and their influence on the initiation of a collaborative management approach. We address the following research questions:

RQ1: How do local actors shape their exchange relations as preconditions for the establishment of a CLM programme?

RQ2: What are the specific requirements of initiating a CLM in the investigated case, and how can a transdisciplinary approach support the process?

RQ3: Which general conclusions can be drawn for similar cases at the intersection of agriculture, nature conservation and tourism?

There is no commonly agreed definition of CLM. Our understanding is widely congruent with the concept of integrated landscape management as described by Bürgi et al. (2017).The authors operationalise it as a continual and adaptive process of joint learning between multiple stakeholders who co-design and test solutions towards a sustainable landscape development.

## Research Design and Methods

### Case Selection and Access

The study is part of the transdisciplinary research project *ginkoo*, which aims at developing knowledge and instruments to support the management of sustainable land use innovations. Because transdisciplinary research starts with the description of a complex real-world problem, the case of the Spreewald’s traditional landscape was included in *ginkoo* after local actors stressed the problem of land abandonment and the accompanying loss of the traditional landscape and its biodiversity. The project period is five years (2014–2019). The science–practice collaboration was organised by a dual coordination structure: one regional coordinator who is employed at the biosphere reserve and located directly in the region and one scientific coordinator located at Humboldt University in Berlin. Regular meetings, workshops and established communication routines provided particularly good access to the case study field.

### Case Study Design

The research design is based on an iterative research strategy that uses a deductive-inductive approach. We applied the principles of case study research presented by Yin (2018) and the transdisciplinary case study approach (Stauffacher et al. 2006). In close cooperation with actors from science and practice, we began with a comprehensive analysis of the situation following the methodological steps of Clarke (2005). To identify key actor groups and interviewees we conducted initial explorative interviews and applied the ‘snowball principle’ (Reed et al. 2009). Subsequently, we developed an analytical framework derived from a literature review on the pre-conditions of collaboration and co-management of natural resources. The resulting deductive categories roughly guided our data collection and analysis as sensitising concepts. During the process of analysis, we were interested in identifying additional inductive categories, which were derived from the material following the principles of open coding.

### Analytical Framework (Preconditions for Successful Collaboration and Co-management)

As outlined above, empirical generalisations with regard to the management of land and natural resources are difficult due to high context-specificities, a large number of interacting variables and variances among different cases (e.g., Ostrom 2001, Cox et al. 2010). In the scientific literature on collaboration and the collaborative management of natural resources, one finds a multitude of principles and factors that influence the success of collaboration (Almeida et al. 2018, Dania et al. 2018, Evans et al. 2011).

Some frameworks consider collaborations as passing through different stages of ‘maturity’ (e.g., Jamal and Getz 1995, Nölting and Schäfer 2016), where different factors play a more or less important role at different times. However, most case studies address established resource use systems. Less empirical evidence is available on factors that are especially important in the initial phase of a CLM programme. Assuming that the cooperation under investigation is in the initiation phase, we focus on analysing the preconditions of a successful collaboration. In addition, we start from the assumption that collaborations are socially embedded and highly dependent on actor-specific relationships, communication, and mutual trust (Pinkerton 1989).

We identified the following frequently mentioned categories that can be used to describe and analyse actor relationships during the initial phase when collaboration is being established (Gray 1985, 1989, Jamal and Getz 1995, Plummer and Fitzgibbon 2004): (i) actors and groups of interests; (ii) problem awareness; (iii) problem definition; (iv) actors’ interrelations; (v) main interest and value-based objectives; (vi) existing networks and willingness to cooperate; and (vii) needed resources to convene and enable collaboration.

### Data Collection and Analysis

The results are based on the analysis of empirical data from different sources. We conducted and transcribed semi-structured interviews with 25 representatives (farmers, small land owners, nature conservationists, tourism providers, and a political representative), collected and screened articles from the local newspaper, used reports and protocols from workshops and websites, and participated in numerous events such as workshops, informal talks, and local field trips. The interviews, documents (protocols and reports) and field notes were analysed and interpreted following the guides to qualitative content analysis of Mayring (2014) and Kuckartz (2012). Data processing was performed using the software MaxQDA. Interviews were coded and case summaries authored, and subsequently cross case conclusions were drafted (following the recommendations of Yin 2018 and Kuckartz 2012). Table 1 provides an overview of the interviewees. Quotations (Q) that prove and illustrate results of our analyses can be found in the Supplement. References on Quotations are numbered and complemented by the acronym of the interviewees’ actor group (Qn_Acronym).

**Table 1:** Overview of interviewees

Actor group	Number of interviewees	Acronym
Member of biosphere reserve	2	BR
Tourism expert	1	TE
Farmers’ association (representative)	2	FA
Nature conservationist	2	NC
Farmer	7	F
Local politician	1	P
Tourism provider	2	TP
Land owner	8	LO

## Results of the Case Study: The Historical Cultural Landscape of Spreewald (RQ1)

### Case Study Background and Setting

**Fig. 1:**
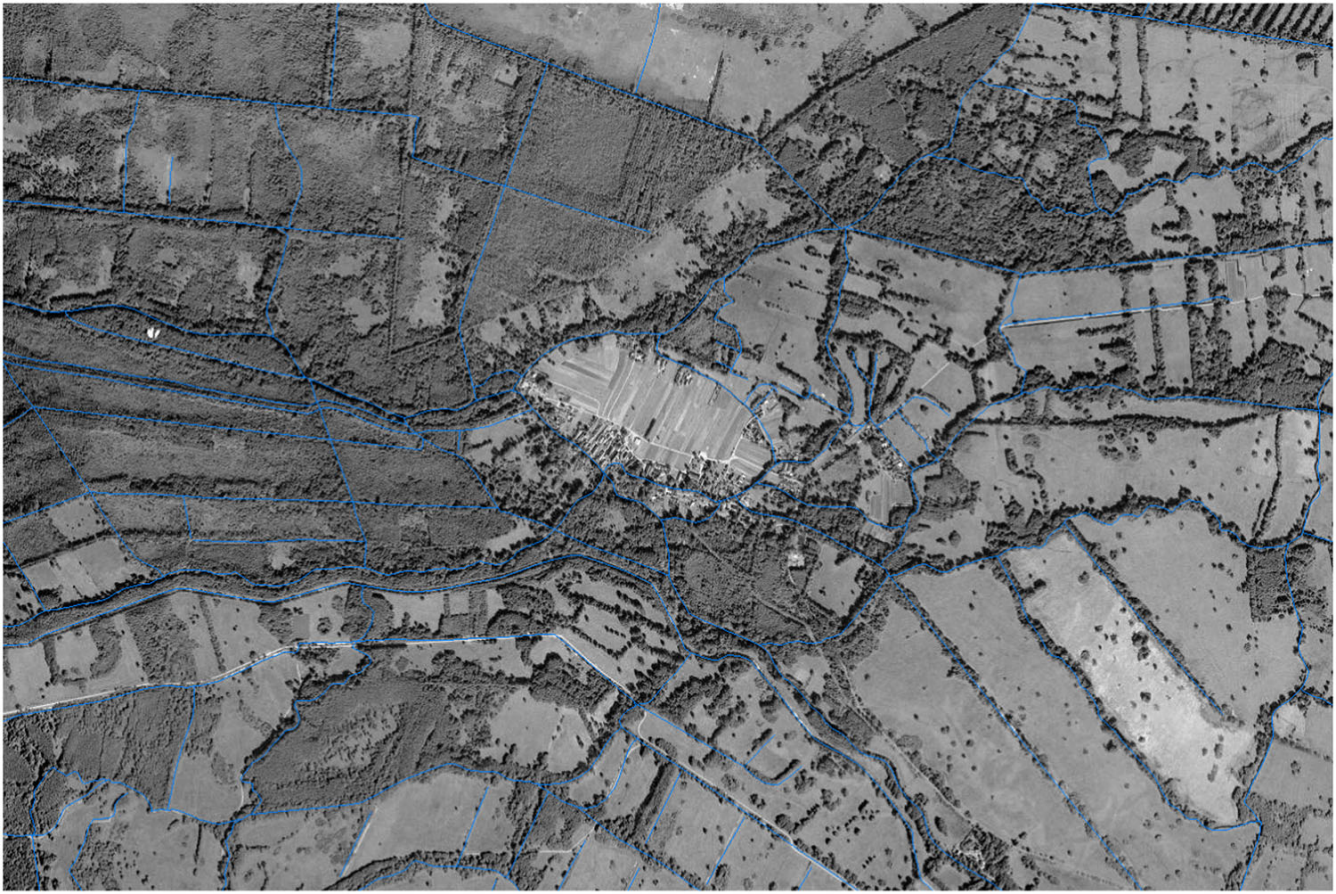
Satellite image of a typical part of the case study region (known as Spreewald biosphere reserve) located in Northeast Germany close to capital Berlin. The region is characterised by a small-scale structured agricultural landscape

The Spreewald region, located southeast of Germany’s capital Berlin (see Fig. 1), is a flood plain characterised by its distinctive cultural landscape, which consists of a broad network of water channels, open marshes (including water hammering wetlands), floodplain forests and small-scale woody plant elements (water channel margins and hatches). These conditions result in high habitat and species diversity.

Increasingly, the wetlands typical of the region can no longer be cultivated and managed profitably. Due to the high moisture and small scale, many meadows require a manual mowing. In addition, many sites are only accessible by boat. As a result, more and more land is being abandoned, and there is a serious threat that it will be released from utilisation in the future. In many areas, the process of natural succession (growth of sedges and reeds, as well as reforestation) has started, and the biosphere reserve estimates that ~1500 to 2000 ha are already affected.

Hence, the traditional landscape is about to lose its typical half-open scenery, with unfavourable consequences for biodiversity conservation and landscape-aesthetic aspects, both of which are important for regional identity and tourism.

Due to the lack of financial resources for sustainable landscape management and the preservation of the open landscape, local actors from nature conservation, agriculture and tourism are looking for innovative solutions to support the maintenance of the typical historical cultural landscape. Thus, interviews and talks revealed that several collaborative innovation processes had been initiated in the years before this study was launched. These processes aimed to maintain the cultivation of the cultural landscape through actions such as the thermal use of hay, the use of donor instruments to involve tourists, and land pooling for more effective conservation measures.

The declared aim is to merge several partial solutions and local initiatives into an integrated, innovative and systemic strategy and maintenance concept for the traditional cultural landscape of the Spreewald region. This goal presupposes collective action and collaboration between key actors.

### Actors and Groups of Interest

At the time of analysis, the idea of a collaborative integrated landscape management that involves local actors was still in its infancy. To support this idea, the civic foundation ‘Cultural Landscape Spreewald’ was formed in 2007. Initialised by different societal actors from the public but also from the private sector the aim of the citizen foundation is to preserve the very unique landscape in the Spreewald regions with all its typical landscape elements. Amongst the founders one can find a range of regional municipalities, private associations like the regional tourism association as well as local firms and individuals. As shown in Table 2, we identified four main actor groups which are of special relevance for the development of a CLM: the biosphere reserve management, farmers and land users, tourism providers, and local residents (including small landowners who do not use their land).

**Table 2:** Identified actors and groups of interest (results from the interviews)

	Biosphere & nature conservation	Farmers	Tourism providers	Local residents & small land owners and users (often mute actors)
**Problem awareness**^a^	High	High	Moderate/partly high	High/partly unknown
**Problem definition**^a^	Loss of areas with high biodiversity value (protected species)	Loss of income and agricultural land	Loss of attractive scenery	Loss of attractive scenery
**Main interest and (value-based) objectives**^a^	Biodiversity & nature conservation	Income and cost recovery	Attractive scenery as a basis for tourism	Maintenance of cultural landscape and heritage
**Willingness to cooperate**^a^	High (initiating and driving)	Mainly scepticism and conflict; mistrust	Hesitation/rejection (free-rider problem)	Mainly scepticism, in some cases high
**Needed resources to enable collaboration**^a^	Time, manpower, facilitation skills	Time, manpower, capacity to collaborate	Direct or indirect payments, broker skills	Time, property rights (in case of landowners), capacity to collaborate
**Additional side-benefits**^b^	Communication: information on processes in the BR; increased acceptance and importance, trust-building	Conservation of cultural landscape, farmers’ image (biodiversity and nature conservation)	Marketing effects (image of ‘responsible tourism’)	Cultural identity; economic strengthening of region, recreation

^a^Based on analytical framework (see 2.3)

^b^Inductively derived

A major promoter of the civic foundation was the biosphere reserve management. The biosphere reserve has the overarching goal of harmonising biodiversity conservation and regional human activities. In addition, several local actors are affected by land use abandonment due to their different main interests. Local farmers are losing income, and landowners potentially cannot recover their running costs (taxes). Local residents stated a high commitment to and interest in preserving the typical landscape because it significantly contributes to regional identity. Furthermore, the tourism sector is one of the main beneficiaries of the region’s attractive landscape scenery, as there is strong potential for more than 1.8 million overnights per year.

### Awareness of the Problem and Its Definition (from the Perspective of Different Actor Groups)

The interview analysis shows that all actors are aware of the problem of gradual change to the landscape. Different actors report that they have been observing this transformation over a period of three decades and that it began to be more pressing with the shift from the socialist planned economy to a market economy in eastern Germany. As a result of that shift, many farmers abandoned their businesses and agricultural plots started to run wild.

Despite a common awareness of a ‘crisis’, the way in which the perceived problem is defined still differs (see Table 2). From the perspective of the nature conservationists, the most important and urgent problem is the loss of areas with high nature value (especially the threat to protected species). The conservationists’ major aim is the protection of nature and biodiversity; they argue that these attributes are cross-sectorally valuable for conservation as well as for tourism conducted from a cultural history perspective. In short, nature and biodiversity together constitute a ‘unique selling point for the region’. Interviewed nature conservationists emphasise that in this case, the aim is not land use restriction, but, on the contrary, the preservation of land use.

In this regard, the conservationists’ concerns intersect with the main concern of farmers, who define the problem as a loss of agricultural land. The farmers’ aim is to maintain the agricultural sites and the opportunity to cultivate them, which is irretrievably lost, or—from an economic point of view—hardly recoverable, once the land is abandoned. The farmers consider it their responsibility to take care of their property. However, even if they emphasise their needs for economically rentable land use and for cost recovery, they also state that they do not want to become mere caretakers of the landscape without the production of food and fodder; instead they want to continue as cultivating farmers. They fear that in the future, the problematic sites might be managed by only one distant, non-regional enterprise. However, the farmers are not unanimous in their opinions: one farmer who advocates organic farming regards structural transformation in rural areas as the main problem. From his point of view, small farmers are increasingly replaced by large agriculture holdings that have no interest in soil and nature or in landscape conservation.

Representatives from the tourism sector report that the Spreewald, based on its appearance today, is perceived as the economic basis of tourism (Q1_TP, see Supplementary Data). The protection and conservation of landscape scenery is perceived as a central issue, with the landscape and the experience of nature, it provides, being crucial. Thus, tourism providers observe the transformation of the landscape with deep concern (Q2_TE).

### How Do Local Actors Shape Their Relations (Interdependencies in Terms of Mutual Expectations and Perspectives)?

Interviews reveal a high potential for conflicts between nature conservation and land use interests. The management of the biosphere reserve is largely perceived as a threat responsible for land use restrictions and inadequate water management. Thus, a multitude of prejudices and a pessimistic attitude towards the biosphere reserve have been reported. For example, one interviewee (TE) illustrated his opinion by recalling the paradox that a ferry operator reported to him: some fauna species have nearly died out since the biosphere reserve was established in the region.

In an interview, one farmer also expressed great disappointment with the biosphere, which is accused of placing nature conservation above everything else (Q3_F1).

The representative of the biosphere confirms that he does indeed face this negative atmosphere. He describes situations with local actors as ‘combats’ that are characterised by strong aggressiveness and defensiveness. This is attributed to frustration resulting from radical social change after the end of the German Democratic Republic (GDR) (Q4_F2). Discussions are also very emotional and sometimes also irrational or non-objective, and it is common to blame the BR as a ‘scapegoat’ (=’Sündenbock’) (Q5_BR, Q6_F2).

In this context, it is also documented that land use restrictions and current water management led to perceptions of paternalism. Remarkably, the theme of ‘conflict’ was prevalent throughout the interview with the biosphere reserve representative, even though that theme was not introduced in the form of a question.

Another perspective relates to the farmers. Nature conservationists and some small farmers criticise the advancing structural change, which entails a loss of small-scale farmers and an increasing concentration of land owned by only a few large agricultural companies. These changes are resulting in a lack of responsibility for the environment.

Tourism providers view farmers with indifference; their dependency on farmers’ contributions to landscape maintenance remains largely unconsidered. In this context, the role of knowledge exchange, mutual understanding and communication is emphasised (BR; TE; NC; TP). In contrast, interviews with farmers and nature conservationists consistently reveal expectations that tourism should advocate for and financially support the maintenance of the cultural landscape. However, a tourism expert notes that, contrary to what is commonly believed, tourism is often characterised by low revenue (Q7_TE).

### What Are the Different Actors’ Value-based Objectives for Landscape Development?

When asked about the ‘typical Spreewald’ landscape, the interviewees find it difficult to define this concept concretely (BR, TE, P, F) because cultural landscapes are always in a state of change and are heavily influenced by anthropogenic use. However, actors had different ideas regarding the development of the cultural landscape. Interviewees attribute these differences to different actor demands, which also change over time and generations.

From a touristic perspective, the typical ‘museum landscape’ with haystacks and thatched roofs is increasingly less in-demand, while ‘wilderness’ and ‘pure nature’ are in higher demand. This change in landscape preferences on the part of tourism is concerning to the BR representative, who fears a loss and undermining of the distinctive nature of the landscape and the region (Q8_BR).

While representatives from nature conservation emphasise the maintenance and preservation of biotopes and species with high nature value, the tourism expert notes that tourism providers and nature conservationists differ in their notions of and perspectives on the cultural landscape. From the conservation point of view, the preservation of the open landscape with the typical wet meadows and the associated typical species composition is essential. In contrast, tourism providers describe a ‘narrow landscape’ consisting of river and forest as a typical Spreewald landscape (Q9_TE). Overall, the tourists and the tourism providers are primarily interested in an attractive landscape, in which details played a minor role’ (Q10_TE).

At the same time, the interviews also reflect a critical questioning of the archiving of a mere ‘museum landscape’ (represented by the artificial building of haystacks), which refers to a long-gone, fragmented style of meadow management. This perspective is also shared by some locals who do not want to be part of a ‘real life museum’.

While tourism representatives, BR representatives and conservationists have different ideas for the development or preservation of the landscape, the interview with the representative of the farmers does not reveal any specific conception of the landscape. Rather, the focus is on the management of the land and water resources as well as their consequent economic uses. This focus is also the basis for a concept of sustainability that emphasises the preservation of land use. Against this background, keeping the landscape open and preserving arable land through adequate water management are mentioned as important goals. This objective is shared by many small landowners, who prefer a ‘tidy’ landscape composed of well-cut meadows with tree-lined boundaries—an image that they remember from their childhood (Q14_LO).

### Willingness and Opportunities to Cooperate

At the time of the investigation, some forms of collaborative innovation had already been initiated, aimed at preserving the typical Spreewald cultural landscape. However, these were limited to bilateral and isolated cooperations, and they had a rather random and fragmented character (thermal utilisation of biomass, tourism co-products, tree sponsorships, wet meadow shares, etc.). From the point of view of the BR, these efforts will not be sufficient to protect the specific wet meadows permanently. Therefore, an integrated development concept is advocated, which combines the different sub-solutions and strives for an inter-sectoral and strategic collaboration among tourism, agriculture, small landowners, and nature conservation. With regard to the question of opportunities for cooperation and the willingness to participate in innovation processes, the interviews reveal the following:

The actors consider direct cooperation between agriculture and tourism (as a spin-off enterprise) to be rather difficult to achieve because the agricultural structure in the Spreewald is no longer characterised by small agricultural enterprises (TE) and is increasingly dominated by large agricultural companies. These large farms, which at the same time represent a low level of actor diversity in the agricultural sector, are perceived to lack identity and solidarity with the region. From the perspective of some other actors, their pure focus on profit maximisation neglects issues of nature conservation and land conservation (F, NC).

For a while, there was some discussion of introducing a tourist tax for landscape conservation. However, it turned out that such a general levy would not be accepted by the tourism industry. Attempts to introduce a ‘Spreewald tax’ similar to a visitors’ tax have already failed in the past. Instead, as a tourism provider stressed, landowners must maintain their own land plots and bear the responsibility for doing so (Q11_TP).

There is also concern on the part of tourism providers that a general tax may result in the artificial preservation of a pure ‘museum landscape’. Tourism providers also note the ‘free rider problem’, where some pay while others only benefit (TP, FA). Furthermore, tourism providers have expressed the criticism that the BR initiated many ‘good ideas’ such as tourism co-products but did not involve tourism providers. As a result, co-products are not perceived as adequate (TP).

Trust is noted as an essential prerequisite for cooperation. However, trust is simultaneously described by the actors as being severely damaged and difficult to restore: ‘… *there we come across granite in the Spreewald*’(Q12_P). Above all, the BR is perceived by many actors as threatening and patronising. Here, reference is repeatedly made to the process by which the Spreewald biosphere reserve was designated in the 1990s. The region’s local residents, small landowners, and farmers are frustrated that they had no voice in this designation process. Similar frustrating experiences are recalled with regard to a major regional nature conservation project, which was carried out between 2004 and 2014 (LO).

According to some interviewees, another barrier not only to cooperation but also to the willingness to try new things is seen in the mentality of the actors, who (as a result of their socialisation in the GDR) have very little entrepreneurial spirit. New ideas and projects are often initiated by people from outside. A lack of ‘sense of community’ is noted.

### Resources to Enable Collaboration

Table 2 shows that time, manpower, and facilitation skills are important required resources to coordinate and enable collaboration. Although central tasks of biosphere reserve management are to organise processes that help to preserve and develop the cultural landscape and to harmonise nature conservation with socio-economic demands, the BR management employee of the Spreewald region states that the BR does not have enough financial and human resources to establish and maintain laborious collaborative processes (BR). Moreover, the other actors do not consider BR management to be a trustworthy and legitimate moderator. Rather, the role of BR management is described as that of an outsider in the community (Q13_TE).

Interviews also revealed a two-sided problem: an ageing population and the corresponding lack of a critical mass of engaged and innovative actors with the necessary skills and capacities to collaborate. Moreover, critical actors had only very limited time to contribute; and sometimes they lacked the capacity and trust to collaborate.

## Discussion: Lessons Learnt

The results show that attempts to establish collaborative approaches intended to preserve the typical cultural landscape in the Spreewald region date back almost 30 years. Even if some initiatives were established successfully (e.g., ‘meadows share’, thermal use of hay, tree sponsorships), these projects are still very small and have not had a noticeable impact on landscape change. It is widely acknowledged by local actors from all actor groups that the typical cultural landscape of the Spreewald region is undergoing extreme transformation and is increasingly being lost. As a result, the BR argues that an integrated and inter-sectoral collaboration that includes all relevant and concerned actor groups is needed to develop sufficient and effective power. At the time of this case study, such an integrated initiative was still at the initial stage, despite long-standing attempts and many past efforts on the part of BR.

### How do the Results Relate to Other Case Studies on Collaborative Approaches? (RQ 3)

In addition to identifying a number of implications for ways to improve CLM projects (see 4.2), we found that most aspects that are frequently reported in the scientific literature on collaborative approaches also played an important role in the analysed case study (for an overview see Table 3).

**Table 3:** Preconditions for the development of a Collaborative Landscape Management (CLM) programme identified in the case study and related to evidence from literature

Deductive categories	Sub- categories	Evidence in literature
Actors and groups of interest	• Interests in issue/motivation • Diversity of actors • Commitment • Power and influence	e.g., Nölting and Schäfer 2016, Gray 1989, Almeida et al. 2018, Dania et al. 2018
Problem awareness and definition	• Perceived crisis • Urgency and importance • Responsibilities (ownership)	e.g., Gray 1989, Plummer and Fitzgibbon 2004, Folke et al. 2005, Biggs et al. 2010, Sotirov et al. 2017
Value-based objectives	• Targeted cultural landscape • Concept of sustainability • Coincidence of or shared values	e.g., Gray 1985, Jamal and Getz 1995, Kenter et al. 2015
Actor’s interrelations	• Perceived interdependency • Mutual expectations and appreciation • Trust • Communication (knowledge exchange, mutual understanding)	e.g., Hulshof and Vos 2016, McGinlay et al. 2017, Gray 2004, Almeida et al. 2018
Willingness to collaborate	• Acceptability of solutions • Free-rider problem • Sense of community • Past experiences and frames • Victim identity	e.g., Trimble and Berkes 2013, Hazard et al. 2018, Goffman 1974, Gray 2004
Resources	• Neutral leadership/moderator • Time • Personal resources (diverse and innovative actors) • Financing • Skills, competencies	e.g., McCarthy et al. 2004, Fleeger and Becker 2008, Beckley et al. 2008, Cheng and Sturtevant 2012

#### Shared values are especially important when actors’ dependencies differ

One important point is that the awareness that a landscape change with negative implications was occurring led to a perceived ‘crisis’ and ‘awareness of a problem,’ which together served as a starting point for initiating a CLM project (e.g., Gray 1989, Plummer and Fitzgibbon 2004, Folke et al. 2005, Biggs et al. 2010). However, we also found that even when problem awareness is high, the ways in which the problem is defined and framed can vary (Sotirov et al. 2017). These different perspectives can be related to different types of dependency on the ethical values of the landscape (Kenter et al. 2015, Cooper et al. 2016). While farmers are immediately economically dependent on plots and their cultivation, tourism providers tend to have larger tolerances for change. In their business, they depend on visitors’ overall impression of the landscape. Thus, the impacts of landscape change on the incomes of tourism providers remain unclear. This uncertainty might partially explain why tourism providers do not recognise their mutual dependency with farmers, while farmers, in contrast, have high expectations of the tourism sector. We argue that even if some scholars regard the ‘coincidence of values’ as an important precondition (e.g., Gray 1985, Jamal and Getz 1995), at least in cases where dependency on common resources differs, successful collaboration actually requires the deliberative formation of ‘shared values’ (Kenter et al. 2015). Such deliberative learning processes could enhance the ‘recognised mutual dependency’ amongst actors and reduce doubts about the outcome of the collaboration by providing ‘an opportunity to collectively wrestle with difficult questions, particularly when there are risks, uncertainties, and winners and losers’ (Kenter et al. 2015, 97). Still, unequal power relations and low appreciation of others’ motivations may hamper the mutual recognition of values (Hulshof and Vos 2016, McGinlay et al. 2017).

#### Negative past experiences and frames are strong barriers to CLM

Another strong barrier is related to the ‘past experiences’ of the actors. The results have shown that past experiences in the case under examination were shaped by the radical social transformations after the end of the GDR. These transformations not only required adaptation to a completely different economic system but also resulted in perceived individual disadvantages. In this context, the radical social change was concurrent with the designation of ~10% of the former GDR as a protected area (see Wegener 2016). The results revealed that ‘historical mistrust’ and ‘victim identity’ linked to a lack of participation in former (landscape) development were prevalent amongst local actors (Gray, 2004). Mistrust is generally seen as a major barrier to establishing collaboration (Almeida et al. 2018). Gray (2004) has shown that the frames of decisive role actors (often resulting from mistrust) have enormous influence on the success or failure of collaborative processes. According to the concept of framing (Goffman, 1974), frames can be understood as inter-subjectively constructed and selective but nevertheless coherent narratives used to make sense of a complex situation. Grounded in individual or collective experiences, knowledge, and perceptions, the framing process is the basis of actors’ argumentations and actions. As reflected in our results, such frames (e.g., mistrust concerning water management practices and regulations) and stereotypes (e.g., nature conservationists as paternalists) appear to be prevalent. Similar results were reported by Hulshof and Vos (2016), who analysed the role of frames as ‘diverging realities’ in Spanish water management.

#### Financial and institutional support is critical for initiating CLM

Also critical when trying to establish and manage collaboration well over time are financial and institutional constraints (Biggs et al. 2010, García-Martín et al. 2016). Institutional support, which makes possible the everyday tasks of an institution (e.g., personnel management, finance, planning), is one key factor that enables the coordinator of a collaboration to function effectively (Biggs et al. 2010). In the Spreewald case, not all actors recognised that such coordination tasks need adequate and permanent resources. Thus, it is crucial ‘to educate and train society about the importance of collaborative management of landscapes’ (García-Martín et al. 2016, 52) and consequently to provide sufficient time and funding for such management.

### Critical Needs and Outcomes of Collaborative Landscape Management (RQ 2)

We identified a number of critical shortcomings that can potentially explain the ‘unsuccessfulness’ of past attempts. In addition, we will show how these challenges can be effectively tackled by applying a transdisciplinary process.

#### There is a lack of an integrated and joint problem definition

The results show that all actor groups could potentially benefit from collaboration aiming at landscape preservation and development. Even if actors’ demands differ (see Table 2), they are all connected with and can be addressed through landscape preservation. All interviewed actors reported a strong interest in the cultural landscape.

In accordance with Gray (2004), we interpret this as a circumstance that increases the likelihood of a successful collaboration. There is not only a widespread perception that landscape change is inducing a crisis but also a recognition that the problem cannot be solved by a single actor (group) (Faehnle and Tyrväinen 2013, Scherr et al. 2013, García-Martín et al. 2016, Head et al. 2016, Almeida et al. 2018).

Although we found a common fundamental awareness of the problem amongst all parties, the way in which the problem is defined by different actors and actor groups varies. The findings also indicate differences in underlying normative goals and values. The awareness of mutual dependency and expectations is partly misaligned and rather low.

A comprehensive and joint framing of the problem, which can lead to a systemic understanding (systems knowledge) involving all relevant actors from different actor groups, still has not taken place. However, this type of framing is widely reported as a central success principle in collaborative multi-actor processes (e.g., Lang et al. 2012, Trimble and Berkes 2013, García-Martín et al. 2016, Foley et al. 2017).

#### A joint vision for future landscape development is needed

This divergence in problem framing corresponds with the lack of a commonly shared vision of future landscape development. All parties had serious difficulties describing what constitutes the typical cultural landscape of the Spreewald region. Cultural landscapes and their preservation as well as development are strongly connected to ethical values and normative goals. Thus, a discussion of common goals appears to be recommendable to integrate all relevant perspectives and to provide knowledge and legitimacy for future action (Plummer and Fitzgibbon 2004, Scherr et al. 2013). Other case studies have also shown that collaborative goal setting and co-design processes led to increased problem awareness (Biggs et al. 2010), shared knowledge (also values) and a generally stronger appreciation of the cultural landscape (Biggs et al. 2010, Plieninger et al. 2013). One part of such a collaborative goal-setting process can be a ‘reframing of perspectives’ in terms of changing negative, blaming frames into a common value-based frame of integrative landscape management (cf. Biggs et al. 2010).

#### The successful initiation of a CLM critically depends on an as legitimately perceived coordinator

A crucial issue that came up in the course of the transdisciplinary process is the question of who can act as an adequate moderator and/or coordinator. Normally, it is a central task of biosphere reserve management to organise processes that preserve and develop the cultural landscape and to harmonise nature conservation with socio-economic demands. However, due to a reported lack of financial resources, the BR is unable to take on this role effectively in this case. The results also show heavy mistrust towards the BR, as it is seen as placing nature conservation above other aims. Thus, BR management is not perceived as a ‘legitimate convenor’. As is known from earlier studies and meta-analyses, the initiator of a collaborative innovation process has ‘a critical impact on its success or failure’ (Gray 1989). In the case under examination, the recognition of mutual dependency is still rather low, and values are not congruent; thus, a ‘neutral third party’ is regarded as the most appropriate coordinator (ibid.). Even if the civic foundation known as ‘Cultural Landscape Spreewald’ might be an appropriate coordinator in the future, at the time of analysis this choice was critically questioned because that organisation’s member structure reflected rather ‘old established networks’ of the region, including BR. Thus, the risk was quite high that some actors would question the legitimacy of the community foundation and withdraw from the collaborative process.

#### Developing ‘collaborative capacity’ amongst key actors is a central success factor

As a central success factor for developing collaborative resource management and sustaining organisational structures, processes, and strategies, many scholars have emphasised the importance of the ‘collaborative capacity of a community’ (e.g., Jamal and Getz 1995, McCarthy et al. 2004, Fleeger and Becker 2008, Beckley et al. 2008, Cheng and Sturtevant 2012). Beckley et al. (2008) define ‘collaborative capacity’ as ‘the collective ability of a group to combine various forms of capital with institutional and relational contexts to produce desired outcomes’. One central indicator is a ‘civic culture’ expressed by local citizens who ‘meet, discuss, exchange, and accomplish tasks in the public sphere’(ibid.).

The results have shown that collaborative capacity—especially with regard to social capital—can still be improved in the case study region. Trust-promoting activities are required, as are competencies in conflict management, improvements in communication skills, knowledge exchange, social learning, mutual understanding and appreciation, etc. (Cheng and Sturtevant 2012, García-Martín et al. 2016, McGinlay et al. 2017, Almeida et al. 2018). Additionally, structures, rules and strategies for CLM still need to be developed in the Spreewald region.

#### Co-production and co-innovation processes can improve outcomes and success

As the results have shown, there is already a series of different partial solutions based on cooperation (e.g., ‘meadows share’, thermal use of cut landscape material, tree sponsorships). These solutions were primarily initiated and developed by the BR and the community foundation. Even if these efforts are widely appreciated, results have also indicated that simple ‘obvious’ solutions might not have the necessary acceptance to be applied by a larger number of actors (Busse et al. 2019). To cite an example from the case study, farmers are highly interested in maintaining land use and avoiding land abandonment. However, the mere cost transfer as provided by sponsorships (‘meadow share’) turns them into ‘landscape caretakers’, which contradicts their self-image as producers of agricultural commodities. Another example is the development of tourism co-products (meaning products that can be sold and promoted by tourism providers, returns are used to finance landscape management), which were perceived as inadequate from the perspective of the tourism providers. Given these findings, we argue that co-innovation processes that involve all relevant actors from the beginning may also increase effectiveness and ultimately improve the outcomes and success of CLM projects. Such co-innovation processes can also be beneficial when applying the design principles of transdisciplinary co-design and co-production (e.g., Lang et al. 2012, Trimble and Berkes 2013, Hazard et al. 2018).

In sum, collaborative approaches such as transdisciplinary (TD) processes can constitute a fundamental basis for CLM, as they support the initiation of institution-building and improve relationships between actors, stimulate co-operation and enhance community empowerment (e.g., Trimble and Berkes 2013, Gruber 2010). TD processes provide a platform for communication, negotiation, planning, and conflict resolution (Zscheischler et al. 2018) that substantially supports the development of a commonly shared vision. In addition, a transdisciplinary research project facilitated by external, ideally ‘neutral’ scientists, can serve as an effective ‘interim solution’ (cf. Kauffman and Arico 2014, Scholz et al. 2017). The search for and the building-up of a legitimate coordinator for the future management of the landscape is thus a central outcome of the transdisciplinary process. In addition, transdisciplinary projects can bring in financial resources and additional (wo)manpower through third-party funding to initiate collaboration, balance the lack of resources, and provide leeway for experiments.

## Conclusion

Traditional cultural landscapes are of great interest to a multitude of actor groups. However, these landscapes are at risk of being lost as a result of structural changes in rural areas and consequent land abandonment. It has been recognised that we lack adequate policies to manage the conservation of cultural landscapes. Thus, the question of how to maintain and manage cultural landscapes where economic benefits are not assured has become a priority. In this context, several scholars have emphasised the role and potentials of collective action and collaborative community approaches to sustainable land(scape) management.

The aim of this paper was to better understand how such a CLM could be built up. Based on a case study from Northeast Germany, we place a special emphasis on the social relationships and social mechanisms that exist among actors.

Our results have shown that in the analysed case study, all actor groups could potentially benefit from the initiation of a CLM project. The findings also reveal that (in addition to institutional and structural aspects) human dimensions such as actors’ relationships and social mechanisms play a major—but so far neglected—role.

Our analysis supports the results of other case studies dealing with the pre-conditions of co-management (see Table 2). Thus, the pre-conditions for co-management of resources (e.g., fishery, water, forest) appear to be largely transferable to the issue of landscapes. In addition, we found that (i) shared values are especially important when actors have different dependencies on natural resources, (ii) negative past experiences and framings are strong barriers to CLM, and (iii) financial and institutional support is critical for initiating CLM.

Finally, we note that transdisciplinary processes can support the initiation of a CLM, strengthen actor interrelations, and lower identified barriers.

## Supplementary information

Supplementary information.
