# Supplementary material for: Challenges to Build up a Collaborative Landscape Management (CLM)—Lessons from a Stakeholder Analysis in Germany
Source: Environ Manage. 2019 Sep 25;64(5):580–92. doi: 10.1007/s00267-019-01205-3 (PMC6838031; doi:10.1007/s00267-019-01205-3)
Supplement: Supplementary file 1 — Supplementary information. [file 267_2019_1205_MOESM1_ESM.docx]

**Supplementary Data. Overview of Interviewees and Quotations from interviews**

Table 1. Overview of Interviewees

| Actor group | Number of interviewees | Acronym |
| --- | --- | --- |
| member of biosphere reserve | 2 | BR |
| tourism expert | 1 | TE |
| farmers’ association (representative) | 2 | FA |
| nature conservationist | 2 | NC |
| farmer | 7 | F |
| local politician | 1 | P |
| tourism provider | 2 | TP |
| land owner | 8 | LO |

**Table 2. Overview of Interviewees and Quotations from interviews**

| Quote No + Acronym | German original statement | English |
| --- | --- | --- |
| Q1_TP | *“... warum die Gäste hier in den Spreewald kommen, ist der Spreewald selbst, d.h. die Natur. Und da speziell eigentlich die Kulturlandschaft, d.h. eine in Europa einzigartige Landschaft, die nicht umsonst ja deswegen auch den UNESCO-Schutz genießt und die als Binnendelta der Spree einfach eine Zauberlandschaft ist …*” | *"... why the guests come here to the Spreewald, is the Spreewald itself, the nature. - especially the cultural landscape, a unique landscape in Europe, which is not accidentally protected by UNESCO and which is, as the inland delta of the Spree, simply a magic landscape ...”* |
| Q2_TE | *“ … da wurden Zahlen genannt wie innerhalb von 20 Jahren ist alles vorbei.”* | *"... numbers were mentioned that in like 20 years, everything is over."* |
| Q3_F1 | “... *wir haben ja sehr große Erwartungen in das BR gesetzt, die leider Gottes nicht aufgegangen sind. Denn ich meine, da ging der Naturschutz über alles und alles andere hat man ein bisschen verdrängt…”* | *"... we set great expectations in the BR, which unfortunately did not work out. Because I mean, nature conservation was put above everything and everything else has been displaced a bit ... "* |
| Q4_F2 | *„Man darf das gesellschaftliche dabei auch nicht vergessen. Die Menschen sind ja hier in der DDR groß geworden mit einem ganz anderen System. Auf einmal gab es den Kapitalismus, wo wir jetzt reingewachsen sind. Aber die mussten da von heute auf morgen damit klarkommen. Und das über die Jahre einfach gesehen hat die Leute in Anführungsstriche resignieren lassen ...“* | *"One should not forget the social aspects as well. People grew up here in the GDR with a completely different system. Suddenly there was capitalism that they had to grow into. But they had to deal with it from one day to the next. And that, over the years, has simply made people resign... "* |
| Q5_BR | *„Aus so einer Runde nehme ich jetzt wieder etwas mit für die nächste Kampfhandlung, sage ich mal. Wo wir wieder zur Sache gehen. Die letzte Runde in X., die war kurz vor Handgreiflichkeiten. Das ging richtig zur Sache. Die Leute sind da so etwas von frustriert. Am Ende haben sie angefangen von Honecker bis sonst was zu philosophieren. Es ging um ganz andere Sachen. Ich dürfe da nicht langfahren wegen Euch Grünen... “* | *"From such a round I take something back for the next fight, I say. Where we go back to business. The last round in X., which was close to a scuffle. That went right down to business. People are so frustrated with that. In the end, they started to philosophize about Honecker [last president of GDR]. It was about very different things like: I cannot go there because of you Greens ... "* |
| Q6_F2 | *„ ... Am liebsten würden sie [die Spreewälder] die Biosphäre abschaffen wollen, wenn sie es könnten ... Weil die Satzung der Biosphäre besagt, Erweiterung der Kernzone, Erhalt der Natur- und Kulturlandschaft und da waren noch 2 oder 3 andere Sachen - ich habe nicht die ganze Satzung im Kopf - das einzige was sie geschafft haben, ist die Erweiterung der Kernzone.... Die Kulturlandschaft wurde zu 90% vernichtet seitdem es die Biosphäre gibt.“* | *"... they [the people from Spreewald] would like to abolish the biosphere, if they could ... Because the statute of the biosphere states, extension of the core zone, preservation of the natural and cultural landscape and there were still 2 or 3 other things - I do not have the whole statute in mind - the only thing they have done is the expansion of the core zone .... The cultural landscape has been destroyed to 90% since the biosphere was founded. "* |
| Q7_TE | *“...man muss immer aufpassen, man glaubt immer, dass im Tourismus so wahnsinnig viel Geld verdient wird und das ist natürlich zum Teil auch nicht richtig.”* | *"... you always have to be careful, you always think that in tourism so much money is earned and that is of course partly not right."* |
| Q8_BR | *“... und der Tourismus folgt natürlich diesen Trends in irgendeiner Form. Was ich also als Verlust wahrnehme ist aus touristischer Sicht bestimmt auch richtig, aber ich will wenigsten hinterfragen, ob die Touristiker überhaupt so weit denken, was sie mit ihrem Alleingang da eventuell provozieren nämlich, dass sich dieser rote Faden nicht mehr weiter spinnen lässt in der Region, dass sich da etwas verwässert…”* | *"... and of course, tourism follows these trends in some way. What I perceive as a loss is certainly true from a tourist point of view, but at least I want to question whether the tourism experts even think so far, what they may provoke with their solo run, that this common theme can no longer be spun in the region, that something is diluted ... "* |
| Q9_TE | *“Die meisten Leute sehen in erster Linie die Fließe als das Besondere. Und das ist eigentlich eher die enge Landschaft. Das ist eigentlich gerade die typische Spreewaldlandschaft. Es ist eigentlich genau nicht diese offene Landschaft…*” | *"Most people see the water channels as something special in the first place. And that's actually the narrow landscape. That's just the typical Spreewald landscape. It's not exactly this open landscape* ... " |
| Q10_TE | *“Ich glaube, diese Details, die auch diese Menschen sehen, die sich damit beschäftigen - das sieht kein Touristiker. Das interessiert meist auch keinen Tourist.[...] dem normalen Touristen, dem geht es nur darum, dass er eine schöne Landschaft sieht.”* | *"I think these details, which also see these people who deal with it - no tourist sees. Also, that usually interests no tourists [...] to the ordinary tourist, it is only important that he/she sees a beautiful landscape. "* |
| Q11_TP | *“... Nachfolge in der Landwirtschaft zu halten ist wahnsinnig schwer, weil [...] die Leute ihr Geld verdienen müssen und ihre Familie ernähren müssen. So, jetzt bin ich aber auch kein Freund davon zu sagen, wir sponsorn alles und legen irgendwelche Gelder um und verlangen jetzt einen Euro mehr Kurtaxe, damit sie ihre Wiesen mähen. Weil dadurch verkommen wir dann auch zu einem reinen Museumsdorf* [...] *unsere Gäste sind ja nicht dafür verantwortlich, diese Landschaft zu erhalten. Das muss man schon selber machen. D.h. diese Binnenkommunikation mit den Akteuren, die betroffen sind von Biosphäre natürlich federführend angefangen, über die Landwirte, die hier leben, über die ja, Gesamtbevölkerung eigentlich. Also jeder muss ja seinen eigenen Garten - wir haben ja alle hier Privateigentum.”* | *"... to keep successors in agriculture is incredibly difficult because [...] people have to earn their living and feed their families. Well, now I'm not a friend to say, we sponsor everything and spend any money and now demand a Euro more tax so they can cut their meadows. Because it degenerates then also to a pure museum village [...] our guests are not responsible for preserving this landscape. You have to do that yourself. That means this communication with the stakeholders affected by the biosphere, the farmers who live here, with the total population actually. So everyone has their own garden - we all have private property here. "* |
| Q 12_P | *„… und da stoßen wir bei den Spreewäldern auf Granit. Weil, nun gut, das ist vielleicht nicht das ganz richtige Beispiel, aber da ist die Grenze, wo Vertrauen auch nicht herstellbar ist. Weil das eine Mentalitätsfrage ist…“* | *"... and there we come across granite at the Spreewalds. Because, well, that may not be the right example, but there is the limit where trust cannot be created. Because that's a mentality question ... "* |
| Q13_TE | *„… wichtig ist, dass man sich an konkreten Beispielen überlegt, wie man die Zusammenarbeit gestalten kann. Dafür ist aber, glaube ich, auch mal wichtig, dass nicht da die Biosphäre sitzt, weil die Landwirte sehen sich, glaube ich, nicht als Teil der Biosphäre…. Ich finde, das ist auf dem Papier so schön einfach, aber in der praxis, ist das gar nicht einfach. Da muss man wirklich mal überlegen, weil im Moment wäre es so – da sitzt die Biosphäre und da sitzen die Anderen. Und das ist natürlich keine gute Voraussetzung, um zu überlegen, wie man jetzt eine Kooperation anders machen kann.“* | *"... it is important that one thinks about concrete examples of how to design cooperation. However, I think it is important that the biosphere is not there, because the farmers do not see themselves as part of the biosphere, I believe .... I think it's so easy on paper, but in practice, that's not easy. You really have to think about that, because at the moment it would be like that - there is the biosphere and there the others are sitting. And of course that's not a good condition for thinking about how to do a different kind of cooperation now. "* |
| Q14_LO | *„.. das* [die Feuchtwiesen] *ist ja das, was den Spreewald eigentlich ausmacht! Das ist uns schon sehr wichtig, weil was jetzt im Spreewald hier passiert ist, der ist ja verwildert, sag ich mal. Der ist ja zugewachsen. So kannte ich ja den Spreewald früher nicht. ... Aber das hat mit dem Spreewald, wie ich den noch als Kind kenne, nicht mehr allzu viel zu tun. Da waren die Wiesen gemäht, die waren blank, da stand mal ein Heuschober oder so ...“* | *“…the wet meadows are what really represent the Spreewald. It [the maintenance of the wetlands] is very important for us. What now happens, here in the Spreewald … It has grown wild, I say. It has become overgrown. I didn’t know the Spreewald like that in earlier times ... I still knew from my childhood. At that time the meadows were cut. There were some haystacks …“* |
